# Proposal of a remote education model with the integration of an ICT architecture to improve learning management

**DOI:** 10.7717/peerj-cs.781

**Published:** 2021-12-03

**Authors:** William Villegas-Ch., Joselin García-Ortiz, Milton Román-Cañizares, Santiago Sánchez-Viteri

**Affiliations:** 1 FICA/Escuela de Ingeniería en Tecnologías de la Información, Universidad de las Americas, Quito, Pichincha, Ecuador; 2 Departamento de Sistemas, Universidad Internacional del Ecuador, Quito, Pichincha, Ecuador

**Keywords:** Artificial intelligence, Big data, Remote education, Learning

## Abstract

University education is at a critical moment due to the pandemic generated by the Coronavirus Disease 2019. Universities, to guarantee the continuity of education, have considered it necessary to modify their educational models, implementing a transition towards a remote education model. This model depends on the use of information and communication technologies for its execution and the establishment of synchronous classes as a means of meeting between teachers and students. However, moving from face-to-face classes to online classes is not enough to meet all the needs of students. By not meeting the needs and expectations of students, problems are generated that directly affect learning. In this work, Big data and artificial intelligence are integrated as a solution in a technological architecture that supports the remote education model. This integration makes it possible to identify the state of learning and recommend immediate actions to its actors. Teachers, knowing the variables that affect academic performance, have the ability to change the components of learning or the method used. Improving learning and validating the capacity of information technologies to generate digital environments suitable for the generation of knowledge. In addition to improving the functionality of educational models and their adaptability to the new normal.

## Introduction

Currently, the society is facing the pandemic generated by the Coronavirus Disease 2019 (COVID-19). To control its high level of contagion, governments have declared health emergencies that force people to carry out long confinements (Li et al., 2020). Confinement has changed the way all activities are carried out, both personal and work. What has caused problems of adaptability to the means used or even that organizations are forced to close their activities or businesses, due to lack of resources for the continuity of their activities and businesses. Scientists around the world are working tirelessly in all their areas of expertise to address the problems that this pandemic has generated (Almaiah, Al-Khasawneh & Althunibat, 2020).

Despite the problems that the pandemic represents, there are lessons that allow different sectors of society to evolve and adapt to a new normal (Pregowska et al., 2021). One of these sectors that has been forced to evolve rapidly is education (Bahasoan et al., 2020). Educational institutions have to continue with their learning processes, especially universities that have adopted urgent plans to continue their activities. These plans include the integration of a greater number of information and communication technology (ICT) tools. ICT have allowed in a certain way to continue with the academic development of their students, for example, face-to-face classes were replaced by synchronous meetings, through videoconferencing systems. ICT tools, in the pandemic, have undoubtedly been evaluated in all aspects and have shown that they are tools that must be included in new educational proposals.

Several works that address similar topics, propose the analysis of learning problems under factors such as economics, psychology and educational methods. These works consider the use of surveys to a population of students to determine the impact that these factors have had on their academic development (Bestiantono, Agustina & Cheng, 2020). The results they have obtained have determined that economic factors are the ones that have the greatest impact on learning problems. They have even determined that there is a great risk for universities when registering increases in the probability of student dropout and repetition (Amory & Seagram, 2004). Other works implement data analysis models that include the use of tools to extract data from the sources available to the university to identify the factors that influence student learning (Palmer, Fazzari & Wartenberg, 2016). These works give us a starting point to propose a solution that allows improving educational management with the use of ICT.

In this work, the factors that have been included in previous works are considered, but new additional variables are added, which allow an identification of the factors that influence the academic performance of students (Huda et al., 2017). For this, an ICT architecture applied to a university for the improvement of learning is proposed, where the fundamental core of this architecture is based on the use of data analysis techniques and artificial intelligence (AI). In this way, it is possible to determine the determining factors that influence learning and through AI techniques, knowledge of the analyzed data is generated to present the results that serve as a contribution to effective decision-making in the educational method. With the aim of designing a scalar architecture, in the implementation of the architecture, the resources and data of the students of the engineering degree in information technologies that belong to the university that participates in this research are considered.

The proposed work is divided into the following sections in order to answer the questions established in the study. The materials and methods section identifies the environment and the research problem, in addition, several concepts are detailed that allow generating the method for solving the problem. The next section presents the results obtained from the analysis. The discussion section compares the results obtained in the proposal with similar works, in this way the validity of the method is established. The final section presents the conclusions found in the development of the work.

## Materials & Methods

To develop the method, in this work a review of the conceptual background that contributes to each of the elements that make up the proposed architecture is carried out. In addition, it is important to identify the problem and the environment where the investigation is being conducted. As an initial stage in the development of this research, similar works that contribute to the theme were identified. The identification starts from the search for jobs that include ICTs that are currently applied in education, and which use AI as a main component to improve learning. The first group includes works related to the use of the latest generation technologies for education. On this, many works address the subject from the point of view of online education. In the online education model, there is a natural coexistence with ICT (Hinostroza, 2018). Its development goes hand in hand with the advancement of technology, and the use of Resources ranges from the use of LMS to the inclusion of interactive games to enhance students’ interest in learning. Another axis where new technologies are involved is in academic management, the new platforms seek to integrate tools for adequate and timely decision-making (Fedorenko et al., 2019). To meet this objective, customer relationship management (CRM) are used more frequently in academic management (Fernández-Gutiérrez, Gimenez & Calero, 2020). In the same way, data analysis technologies are the tools with the greatest demand in current education, considering that the education paradigm has changed, focusing on meeting the needs of students based on what they want to learn and how to do it.

In the second group, works that integrate AI into educational systems were analyzed. In these works, it is highlighted that every day the use of artificial intelligence in our daily lives becomes more and more evident (Pattanayak et al., 2019). However, there are still many elements to work and organize before a true implementation of AI in education can be made (Valtonen et al., 2018). Even so, AI is presented as a great advantage and helps when imparting and generating knowledge in educational models that seek comprehensiveness and true transversality of technology and in the various axes of learning (Хакимов, 2020). These papers consider that the rapid development of next-generation ICTs, such as AI and their deployment across various social dimensions, is likely to deepen existing digital inequalities. These are problems that must be considered in the new educational models, for which it is necessary to start from a robust data analysis model.

### Identification of the problem and the environment

University students, due to COVID-19, go through psychological, economic and technological problems that directly affect their learning (Zhou et al., 2020). Universities offer a variety of educational models; however, their main model is face-to-face education. In the face-to-face education model, it places the teacher as the main actor in learning. He defines what students should learn and how to generate learning. In the face-to-face or traditional learning model, direct interaction with the teacher allows them to detect any deficiency in student performance (Gowensmith, Murrie & Boccaccini, 2010). In addition, the teacher, when interacting with students directly, can make decisions that allow them to improve their learning. However, due to the pandemic, universities changed their educational models to a remote education model, where face-to-face classes were replaced by synchronous meetings (Watson, 2006).

Due to the speed with which isolation and quarantines were decreed, the response of the universities had to change their model just as quickly. This means that all educational content and methods are maintained, with the change in the way the classes are run (Sidpra et al., 2020). Similar works have identified that learning is where most of the problems are found, due to disinterest in the subjects on the part of the students and the lack of control on the part of the teacher in remote classes. To cover these deficiencies, it is necessary to cover certain tasks in the role of the teacher with ICT. Another important factor is the method used; in the face-to-face mode everything is focused on the use of resources as part of the teacher’s task.

### Research question

With the identification of the problem and the conceptual background, it is necessary to establish the research questions that need to be answered with this work. The proposed scenario focuses on remote education to which isolation due to the pandemic has led us. This modality requires a lot of effort from the teacher with tools that do not provide the teacher in a satisfactory way. Therefore, it is proposed to define a question that encompasses the educational model of the university that participates in the study and the existing problem. That is, “in a distance education modality, can new technologies such as AI and data analysis be applied to improve learning or even take the next step to a personalized education model?”

### Conceptual background

As a fundamental basis, in this work several concepts are used that provide a clear vision of the elements that constitute an ICT architecture that allows to improve learning.

### Remote education

Remote education is an operational arm of virtual education in real time, in which learning is a kind of experimentation. While Hodges (Hodges, 2013; Alqurashi, 2016) and others argue that virtual education is creating and sharing a robust educational ecosystem. Planned and designed around seven months before being implemented, which is naturally accessed from anywhere and at the right time. Autonomous learning is the central nerve of virtual education. With the use of the Internet and ICT, it is possible to access information contained in blogs, podcasts, emails and other digital environments. For its part, remote education lacks autonomy, limiting itself to the use of a platform and the pedagogical act is consumed with the online participation of the teacher and students. Likewise, the pedagogical art of the teacher in the virtual sphere is efficient, because the entire academic routine is designed; while in the remote realm, Hodges and others argue, “it requires faculty to take more control of the course design, development, and implementation process,” meaning a lot of overtime work for the teaching community (Hodges, 2008).

In addition, during the pedagogical execution, the learning process can unexpectedly be compromised by problems on the Internet, in electronic devices, electric power or the inappropriate use of the platform, making it difficult to remote class. The virtual experience is free from academic straits, as it is programmed asynchronously, focused on the invigoration of meaningful learning. The themes to be deployed in the slightly remote sphere are brief, based on pedagogical dialogue, highly motivating, with emotional pauses, creating spaces for synergy and cognitive relaxation, which the university authorities do not understand. Given the central role of the teacher in remote learning, the student requires permanent accompaniment and must make enormous efforts to learn, according to De Zubiría, Angel & Merani (1999), it is necessary that someone accompany the students. In virtual education, the student is the manager of his constructivist learning and strengthens his discipline, away from the teacher, limited to acting as a tutor or advisor.

### Information security and student privacy

Information security and user data privacy is a priority for the university participating in this study. But, in the country to which the university belongs, there is no established data protection law that all organizations must abide by. However, the university takes European laws and regulations for handling information as a guide. This forces the academic and administrative departments to abide by the university’s own policies. Student data is used purely for academic activities and cannot be accessed or used by people who do not have the authority to do so. The care of the information is in charge of the information security department, who constantly carry out audits in each of the computer systems. In addition, each report issued by the architecture does not present private data of the students or teachers, these are replaced by identifiers. The data generated in the analysis and that are used by the academic monitoring area allow to identify the students, this task is extremely important, to take effective actions and offer a personalized education. However, the people who handle this information accept confidentiality agreements. The data presented in this work have not affected the protection of student data. Similarly, the data that is extracted from the synchronous class system is found in text files and usage reports.

### Method

For the design of the method, it is important to explain what the solution to the problem is outlined. Universities are carrying out a remote education, where there are several problems to maintain an education where the main actor is the teacher. But the model does not provide the necessary tools to maintain the interest and control of the students in such a way that learning is not affected. The architecture proposes integrating ICT in certain areas of the remote education model to provide the teacher with several tools that allow to guarantee learning.

### Remote educational model

**Figure 1:**
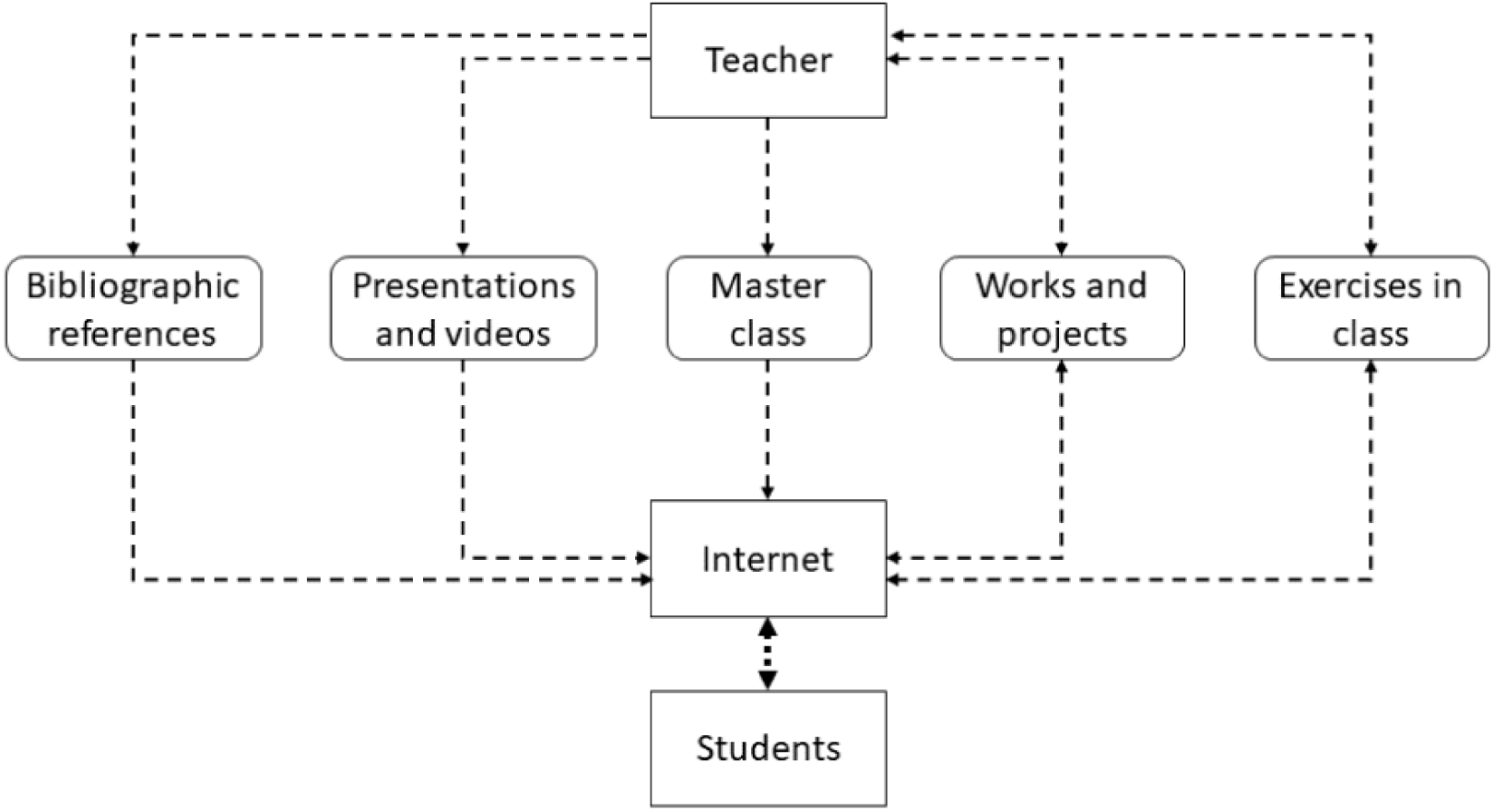
Components of a remote education model.

In Fig. 1, the remote education model is presented, where the teacher fulfills a role similar to that of face-to-face education (Jahng, Krug & Zhang, 2007). Among his assigned tasks, he is the one in charge of managing the bibliographic references that he requires for teaching. He identifies the presentations and videos that the student requires for the mastery of the subject being studied. The main components are the master classes, where the teacher imparts his experience and mastery of a subject in order to generate knowledge in the student (Gowensmith, Murrie & Boccaccini, 2010). To evaluate learning, the teacher is in charge of proposing the different activities or exercises in classes, where the learning result (TLR) to be evaluated is defined. The difference with the face-to-face model is that to reach the student it is necessary to use a videoconference system to generate synchronous meetings.

The figure presents the remote model, which implies the use of ICT, simply as a channel to reach the student through learning management systems (LMS) and synchronous meetings. When using these channels, it is not possible to guarantee student learning (Villegas-ch, Palacios-pacheco & Roman-Cañizares, 2021), the main reasons are, technical problems, student interest, inappropriate remote class method, activities not focused on student needs, *etc.*

### Remote education model architecture

The proposed architecture must be integrated into the educational environment, where it is a priority to start from the problems to be worked on, for which the following list is presented:

• Lack of control in the development of class by the teacher to the students.
• Lack of student interest.
• Non-student centered activities.
• Method used in synchronous meetings.

The listed problems are those that are dealt with the use of ICT in search of learning improvement. The first problem deals with the lack of control in the development of the class by the teacher to the students. This is a problem of synchronous classes, unlike a traditional class, the teacher, not being in face-to-face contact with the student, is not able to detect whether the students pay attention to the class An internet of things model for improving process management on university campus (Villegas-Ch, Palacios-Pacheco & Román-Cañizares, 2020). There are universities that, as a measure, force students to activate the camera during class. This measures little or nothing helps in the control of the classroom environment (Literat, 2015). It implies that the teacher must be more focused on identifying each person through the cameras than on developing the class (Medhat, Hassan & Korashy, 2014). In the technological part, this measure is also ineffective, since in certain cases keeping the cameras on produces detrimental effects in terms of quality of service in communication.

The next problem is the lack of interest of the students during the class, this has been evident since this model has been executed. The evidence is not limited to works that analyze the state of learning, even students have been identified doing other activities and videos have been uploaded to social networks causing trends on the web (Anbang et al., 2017). Therefore, the interest of the student must be worked from an academic point of view with a new proposal in the method of classes. Activities not focused on the student must follow the same line, in the face-to-face mode the use of laboratories with the development of projects guarantees the maximum use of the student’s abilities. The use and generation of virtual laboratories are something that should be included in this architecture.

The method used by teachers in synchronous meetings is another factor that affects learning. This problem must do strictly with the teacher, universities must understand that face-to-face classes and synchronous meetings are not similar, and the way people act in these environments is very different. For example, in online education teachers are constantly trained with techniques that help them cope with synchronous meetings (Tchoubar, Sexton & Scarlatos, 2019). The resources that a teacher handles in the face-to-face mode is different from those that are usually used in online education. Something as simple as a joke on the part of the teacher, can generate greater interest in the class and even the response of the students to this action motivates the teacher to deliver knowledge of it. The charisma or spontaneity of the teacher in the synchronous class does not have the same effect, it can even be something negative for the teacher as they do not find a response from the students (Beldarrain, 2006). Therefore, ICT should be used to generate an ideal space where the teacher feels committed to learning.

### Components of an architecture of a remote education model with the integration of ICT

**Figure 2:**
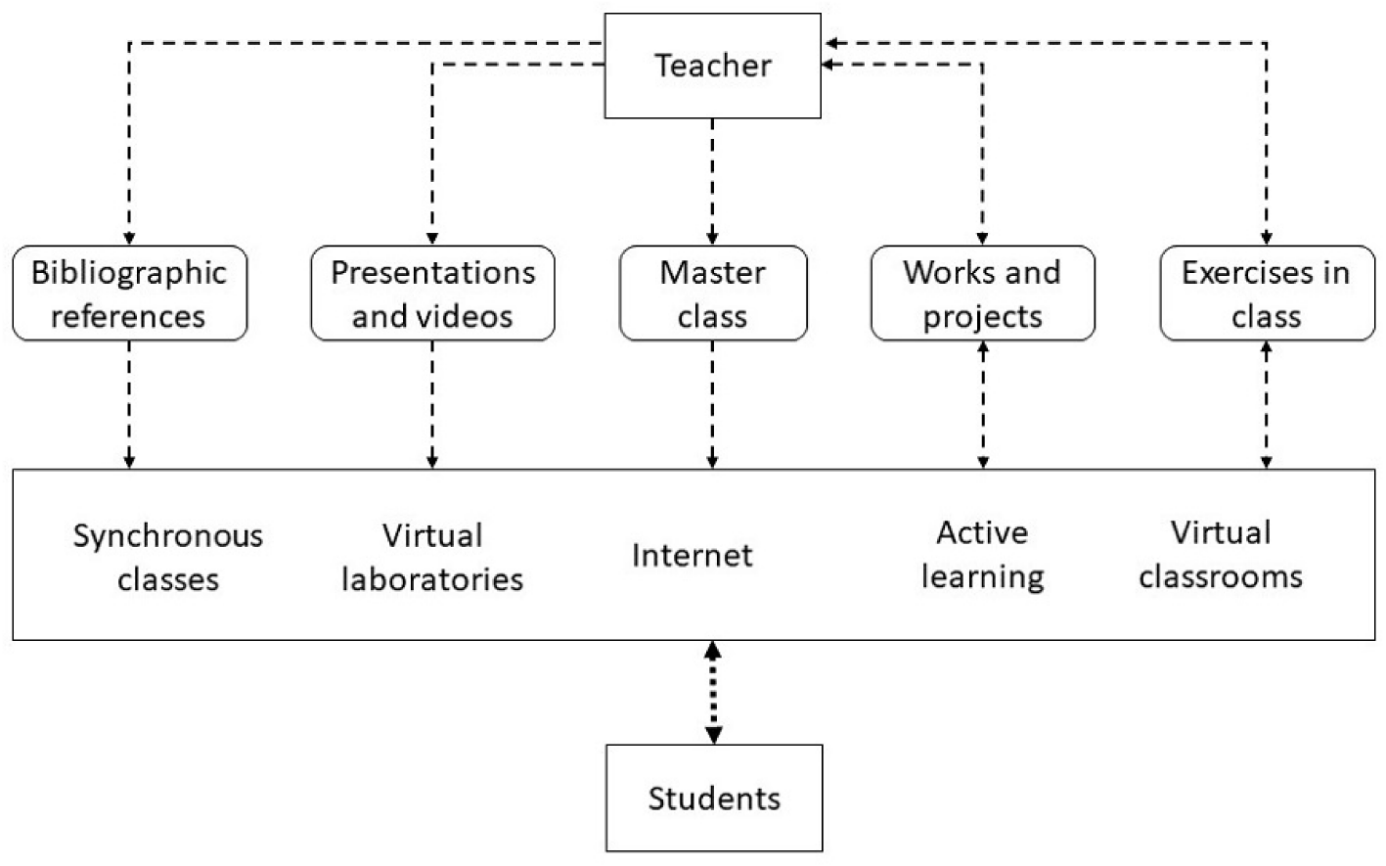
Components of a remote education model with the integration of ICT.

In Fig. 2, the different components of ICT are presented with which it is proposed to solve learning problems. The module where the components have been integrated is in the access defined in the previous architecture such as Internet (Ali, 2020). This layer is where you need to make the greatest amount of changes to integrate ICT to control problems that arise in learning.

### Lack of control in the development of the class by the teacher to the students

In face-to-face classes, the teacher acts as a sensor in charge of detecting any distraction from the students. With the synchronous meeting, the teacher loses this control and the students in most cases have learning problems due to distractions caused by the environment. To avoid this, it is important to establish a set of technologies that assist the teacher in controlling the course (Benshoff & Gibbons, 2011). This qualification is defined in the videoconferencing tool. Universities generally make the choice of the tool, based on costs, the number of concurrent users, availability for generating groups, *etc*. But a more useful factor is audit reports on video calling activities. This information is very important, because it helps to control to some extent that students do not carry out activities that do not correspond to the classes. The tools that present this capacity usually integrate artificial intelligence algorithms that can detect through the peripherals of a computer that the student is active in the synchronous meeting (Sunasee, 2020).

**Figure 3:**
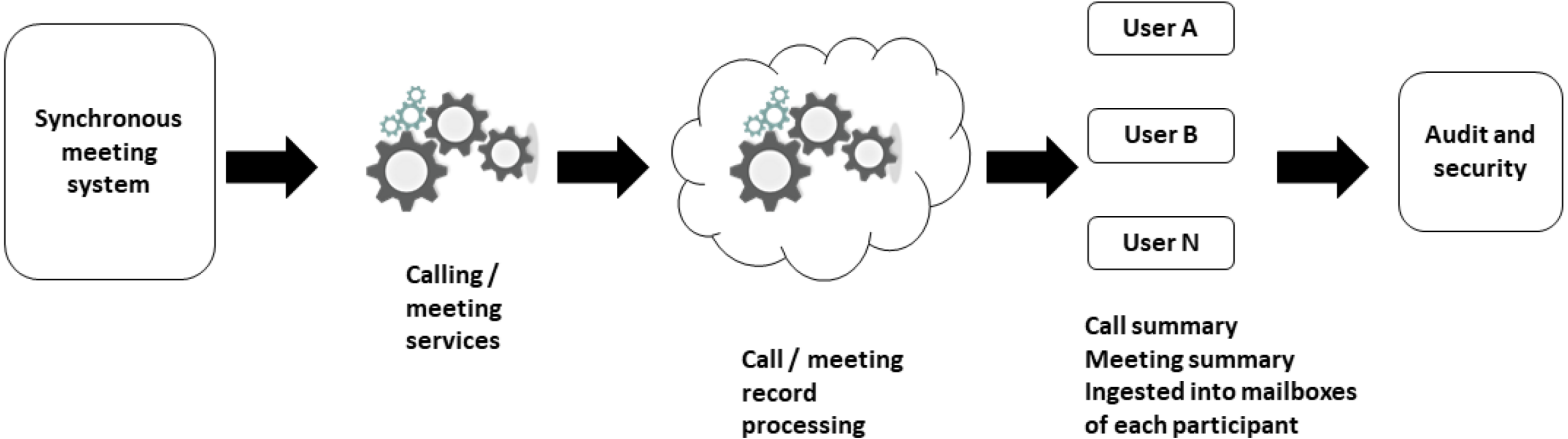
Call and meeting data management flow of a video conferencing tool.

The audits are used to identify students who present a low percentage of effectiveness in the synchronous class. For the proposed architecture it has a greater scope, because it helps to obtain the greatest amount of data to later be processed, obtain knowledge and make decisions that allow improving the control in the class. In Fig. 3, the flow of call and meeting data management that a videoconference system should contemplate is presented. The sense of flow goes from the clients were all the members of the university are. In the next phase is the engine that processes the calls or synchronous meetings previously generated for the different classes. Once the call is established, it goes to the call and record processing phase. This is an advantage that synchronous classes offer, by being able to access the recording of the class, the student can see it as many times as he considers necessary to solve a learning problem. In the next phase, the audits corresponding to each of the participants are generated (Sun, Fukuda & Resch, 2014). Audits are generated in terms of calls, messages or meetings. In the last phase, the information is presented by means of emails or the storage base that integrates the videoconference tool can also be accessed. The information can be extracted to be processed by data analysis tools.

### Lack of student interest

Lack of student interest is common in remote mode, usually because students are not receptive to this type of education. Several studies that analyze the psychological state of the effects of COVID-19 on learning mention that one of the causes of the lack of interest is that students, from the beginning, are not predisposed to continue with a remote education. This factor is compared with students who choose online education, these students have the predisposition to use synchronous meetings as a means of learning (Benshoff & Gibbons, 2011). Even the attitude towards self-education is different from those that due to the pandemic have gone remote (Kerr, 2016). To confront it, it is necessary to create environments where the student generates greater interest in the subject, for this, it is necessary to create virtual environments that contribute to the academic area.

Virtual laboratories or the use of simulators is important in this learning model. But its inclusion requires a job that includes all the learning resources and academic activities. The remote modality, when having the resources and the educational model of the face-to-face modality, does not always have a structure focused on the use of this type of resources that facilitate learning (Ali, Hussein & Al-Chalabi, 2020). The new structure clearly determines that, within the class sessions of each subject, there must be a practical component with the use of simulators or exercises applied in virtual environments. The advantage is that several universities or institutes have focused on creating virtual laboratories, where students can put their knowledge into practice (Hssina, Bouikhalene & Merbouha, 2017). By clearly establishing an appropriate percentage of practices within classes, it forces students to pay more attention and interest to the classes.

### Non-student centered activities

In face-to-face education, the main characteristic is that it focuses on the teacher. This implies that each activity that assesses learning is tailored to what he wants to be learned and how they should do it (Gowensmith, Murrie & Boccaccini, 2010). In traditional education this works because of the follow-up that the teacher carries out in the classroom. In the new modality, it is necessary to modify the way in which each of the activities are generated (Hill & Hannafin, 2001). The best example is the methods used in online education. These methods are flipped classes, strategies for teaching in virtual environments for learning, collaborative learning techniques, *etc*. The change, unfortunately, takes extra time that the teacher must allow for their training (Alqurashi, 2016). It is a necessary time that defines excellence and the improvement of learning.

### Method used in synchronous meetings

This factor does not focus specifically on how the teacher teaches a class; it focuses on where it is carried out. Universities, within their academic quality plans, constantly evaluate teachers. Therefore, the method used by teachers is considered adequate, which allows them to generate learning in their students. When switching to remote mode, many of them went out of their comfort line or even found cases where the use of ICT by teachers was deficient (Totkov et al., 2020). This undoubtedly affected learning, ICT can improve this, by creating digital classrooms. The idea is promoted in the following environment, the universities have generated plans that allow the progressive return to their activities, with students being one of the last groups that will resume their activities completely. Within these plans, it is considered that universities integrate a broader use of ICT in their classrooms (Daniel, 2020). Therefore, there is a limited group of students who will be able to enter a face-to-face class, and most will do so remotely. For this, cameras and videoconferencing systems of higher quality are included. In these environments, the teacher returns to a controlled place that allows him to transmit her knowledge in an ideal way.

### Architecture proposal for a remote education model with the integration of ICT

**Figure 4:**
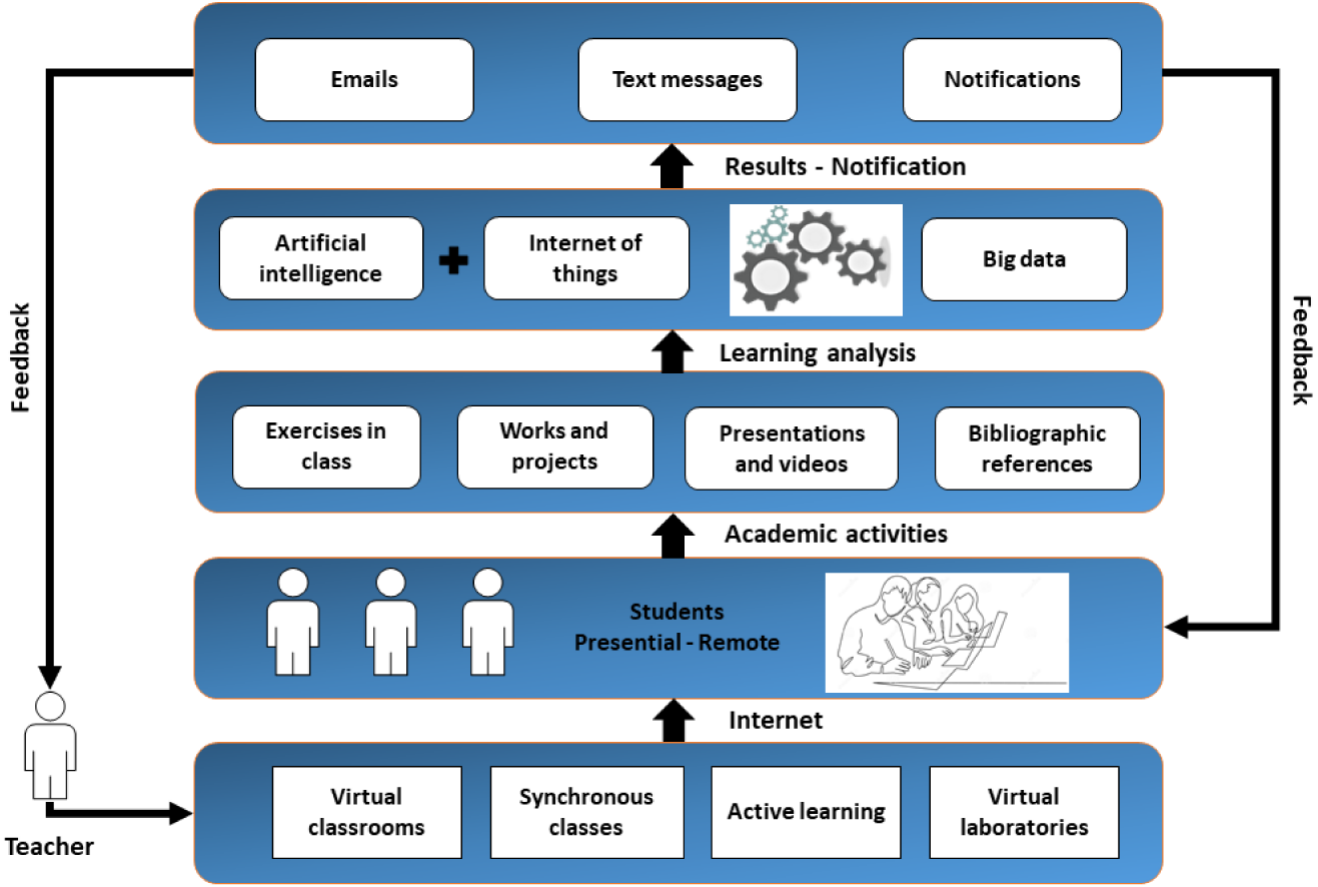
Proposed architecture for a remote education model with the inclusion of ICT.

Once the problems and components that improve the remote education model with the use of ICT have been defined. It is possible to determine a layered architecture, where each one integrates several ICT tools focused on learning. In Fig. 4, the proposed architecture for a remote education model is presented (Akopova et al., 2019). Architecture maintains the teacher as an actor through which all learning passes, this is important to define because it is not proposed to define an online or virtual education (Salmerón-Manzano & Manzano-Agugliaro, 2018). The proposal is based on the face-to-face model.

In the lower layer all the components are presented to improve the problems that have been identified above. The teacher is no longer alone with videoconferencing tools. On the contrary, ICT allow the creation of digital classrooms, generating for the teacher an environment that is closest to face-to-face classes (Gowensmith, Murrie & Boccaccini, 2010). This layer includes video conferencing systems and cameras that detect each classroom and laboratory space. The latter being the ones that have priority in integrating with ICT. The system for generating synchronous meetings is a contracted service. This requires that, in addition to the technical characteristics, it is necessary for the system to offer complete audits, both at the service level and at the user level during the synchronous meeting. The activities proposed by the teacher in this architecture are generated by an active learning model. To do so, the training of teachers by the university is necessary (Brown & King, 2000). These activities must be complemented with the use of virtual laboratories or simulators.

In the next layer are the students, by having classrooms and laboratories that integrate ICT, it is possible to propose a gradual return of the students. In a first stage, universities must choose a small percentage of students to resume their face-to-face activities. To meet this requirement, a percentage of hours that must be met in this mode must be assigned. For example, it is possible to determine 10% of the sessions that must be face-to-face, and the rest are followed remotely. In another group are students who are kept in remote classes.

In the next layer are all the activities that students must do. Each activity now responds to an active learning model; therefore, the evaluation mechanisms are varied. The exercises in class are carried out by means of simulators where each conceptual element is reinforced with the practical part. Other activities that the student is requested to do are the generation of videos and presentations on the different topics reviewed.

The learning analysis layer is the strength of the proposed architecture, by integrating many ICT tools, it is possible to obtain large volumes of data. Data becomes the most important resource that is obtained from each activity that students carry out. Through data analysis it is possible to identify the strengths and weaknesses of students. With this information, decision-making is facilitated to the point that learning can be personalized. In this layer, the largest number of emerging technologies can be added, for this work machine learning (ML) with artificial intelligence (AI), the Internet of Things (IoT) and Big data have been considered (Boiy & Moens, 2009; Madakam, Ramaswamy & Tripathi, 2015; Villegas-Ch, Palacios-Pacheco & Luján-Mora, 2019b). The expected operation of each component is available in the IoT through cameras available in classrooms and other sensors available on campus that provide information about students. ML works in conjunction with Big data to establish academic recommendation and monitoring systems. In addition, there are traditional systems that are in continuous generation of information such as LMS, academic and financial systems (Verma & Pandey, 2016). All the volume and variety of data is processed by a Big data framework that is responsible for generating knowledge of the data.

The notification and results layer are responsible for presenting the knowledge to those in charge of academic quality, to teachers and students. The information is presented in various formats depending on the area to which it is directed. With the correct information on student performance, the teacher can improve the activities or topics, even change the method of the classes (Lin et al., 2021; McHugh et al., 2018). The feedback of the architecture towards the student is marked as an alert, the analysis system tracks the variables that intervene in their learning.

### Learning analysis

**Figure 5:**
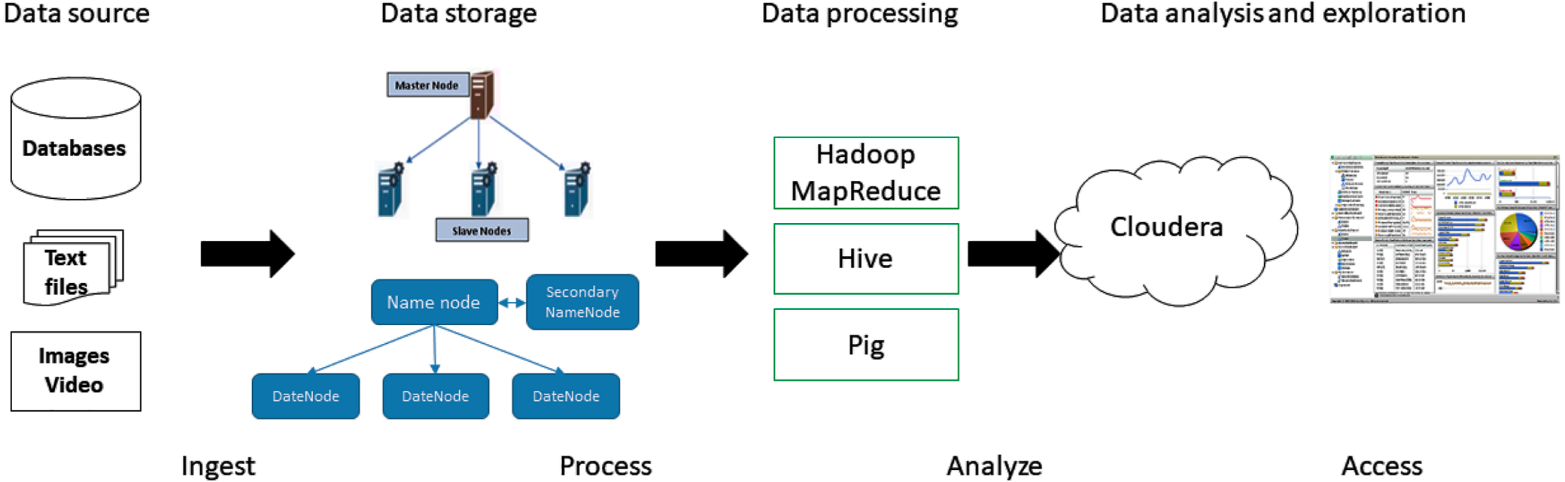
Architecture of a Big data model implemented on Apache Hadoop, for educational data analysis.

Learning analysis has two main components, its operation becomes one of the main axes of ICT architecture applied to the educational environment. It is for this reason that this section has been dedicated to explaining how it works. The educational data generated by the students is analyzed by a Big data framework. The framework developed in Hadoop, presents a cluster architecture with a master node and several slave nodes. The master node is in charge of managing the existing requests and dividing it into threads that are executed in the slave nodes. Therefore, the learning analysis strictly depends on the operation of the Big data framework. In Fig. 5, the architecture of Big data and the components that are responsible for the analysis of learning are presented. In the first section are the data sources. The university includes in this phase, the largest number of sources available on the information generated by students. Common sources include academic and financial systems. Generally, the data found in these systems are structured, in addition, there are sources that store unstructured data. These sources are images, emails, PDF., Videos, *etc*., even though, at this time, sensors and actuators implemented on the campus of the university participating in this study are not used, it is important to consider them when returning to the presence.

 The data is ingested through an Apache Hadoop distributed file system (HDFS). HDFS stores data files on multiple machines to distribute the information to different devices or nodes for processing. MapReduce, hive and pig are used in the processing stage. These tools allow parallel programming, storage, and SQL-like queries to generate relationships in data and find information about learning. Finally, accessing the analysis through Cloudera, the presentation focuses on giving details to the learning actors about the state of the academic performance of each student.

**Figure 6:**
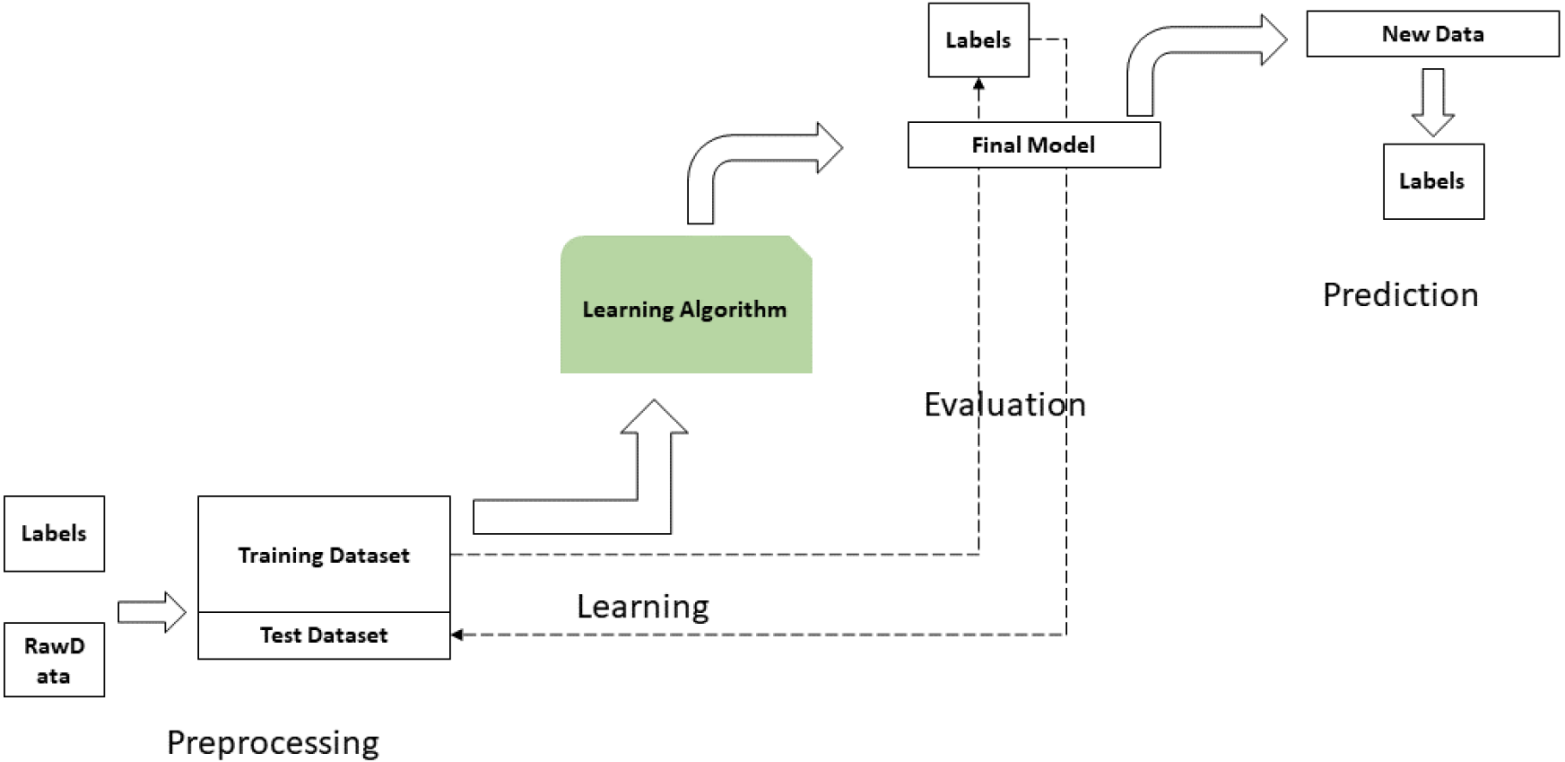
Architecture of a machine learning model, applied to the identification of the state of learning in a university environment.

The result obtained by the Big data framework is consumed by the ML model. In Fig. 6, the architecture and components that allow generating the academic monitoring of each student are presented. The processing phase is one of the most important steps, usually the data is presented in non-optimal formats to be processed by the model. In the learning phase, the algorithm used for ML is the Bayesian classification. Its purpose is to establish a model for the relationship between a certain number of attributes and a continuous target variable. The results obtained allow establishing effective strategies for monitoring the educational performance of students. These strategies are translated into computer systems that become personal assistants that present the status of each student’s learning, for example, the system may report a projected unsatisfactory performance. In addition, through classification it is possible to determine the factors that affect the academic performance of a group of students, by identifying existing patterns in their data. This information is used by universities to implement continuous improvement programs at the institutional level.

In the AI phase, the information collected in the data sources is pre-processed. Sources include polls, txt files, xlms, databases, *etc.* The analysis includes 12 attributes for classifying the patterns found in the data. The data obtained through surveys use a five-level scale, with 1 being the lowest level, inefficient or none and 5 being the highest level, many or excellent.

• 1: never
• 2: rarely
• 3: sometimes
• 4: almost always
• 5: always

In the learning phase, it begins with the selection of the sample, to obtain greater statistical reliability, a simple random sampling was applied, with 24 computer science engineering students. With the information of the students, the database is built, in this the surveys applied to the students are considered, in addition the academic and financial data such as the average of grades and the economic situation were integrated. The academic average was organized into 5 groups.

• A: average (8–10)
• B: average (<8–6)
• C: average (<6–4)
• D: average (<4–2)
• E: average (<2–0)

For the evaluation phase, a correlation matrix is constructed where the attributes considered in the analysis are added *versus* the dependent variable. The correlation allows identifying the incidence of each dependent variable and establishes which of these is the one that should be analyzed with greater granularity in data analysis and learning supervision.

For the prediction phase, a .Arff data file is included; therefore, its creation is necessary from the attributes that present the highest correlation with respect to the independent variable and is subsequently added to the data analysis software. The data in the .Arff file is analyzed using a Bayesian classifier, considering that this algorithm obtains efficient results with little data. The algorithm identifies the main attributes that influence academic performance, and a decision tree is obtained. A decision tree presents the results graphically, facilitating the detection of the attributes that influence the academic performance of students.

## Results

The architecture is implemented in a university in Ecuador, this university is private, which facilitates the integration of new technologies and educational methods. For the implementation and because it is a proposal that is under evaluation, it was implemented in one of the university’s faculties. The college has eight careers, but it applies to the computer science career. This decision is since both students and teachers have knowledge about the use of ICT. By working with this career, it is possible to reduce the time required in ICT training for teachers and students. The computer science career consists of 250 students, however, as it is a pilot plan that seeks a gradual return to a face-to-face modality, a single course has been considered. This course is the seventh semester; it has 24 students.

**Figure 7:**
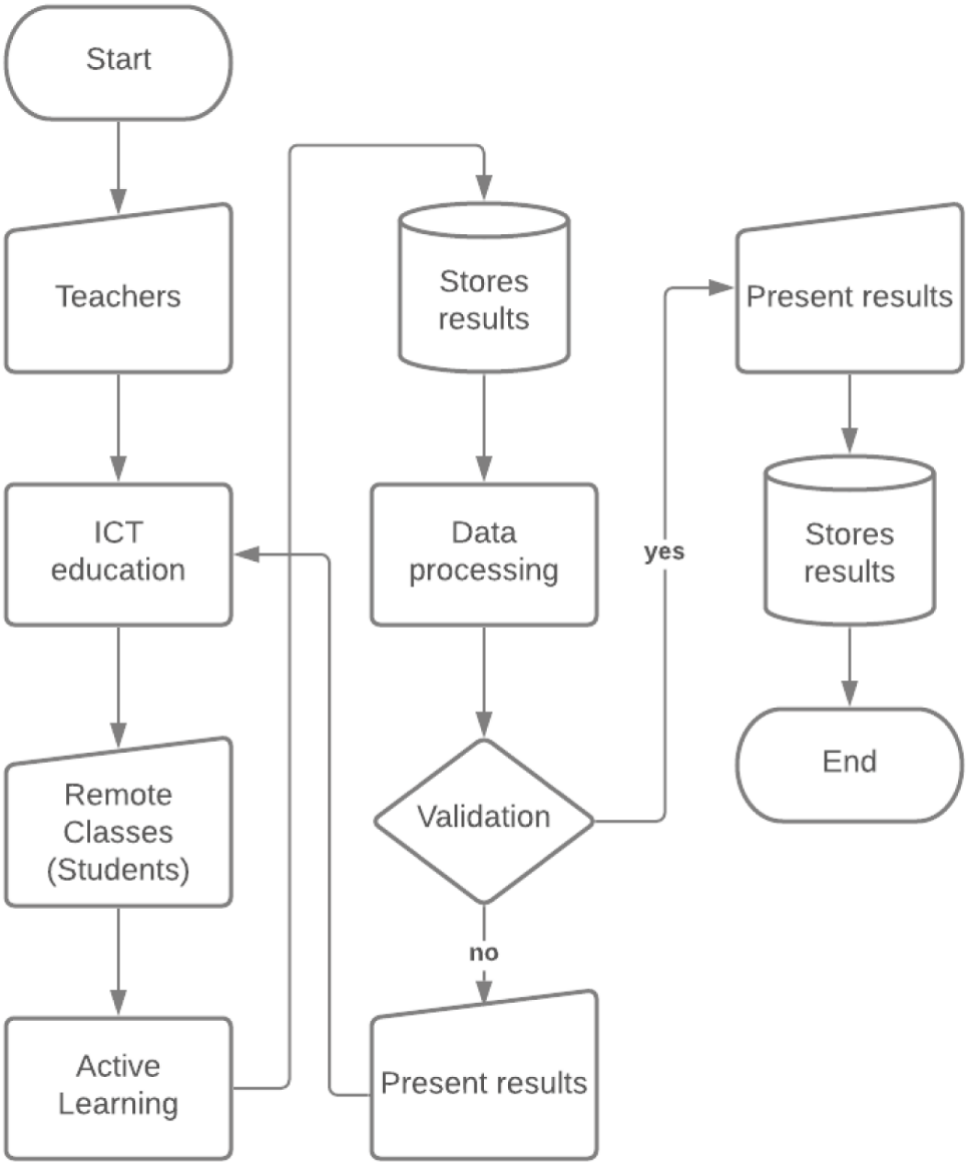
Flow diagram of the operation of ICT architecture applied in a remote education model.

In Fig. 7, the flow diagram of the process with which the evaluation of the method is carried out is presented. The process begins with the teacher entering a class. For the classes, use is made of the laboratories, where a videoconference system was integrated. This system is linked to the software used for the synchronous meeting. In addition, in the classroom there are five students who respond to the 10% of the students who are registered in the subject of networks I. The students who attend the laboratory have been selected according to their age, health status, exposure to COVID-19, *etc*. The data were collected through surveys. The rest of the students continue in the remote mode, and the class takes it, through synchronous meetings and with the use of simulators. The difference and the advantage that exist so far is that the teacher, being in a traditional classroom, returns to an environment that he already dominates and where he can use the greatest number of tools available for the development of his class. In addition, since the class is transmitted using the videoconference system, the teacher is detached from any technical or connection problem. The teacher focuses on organizing their classes or laboratories and carrying out academic monitoring of the students. The laboratories of the subject have network equipment that have a simulator of the same line of equipment, therefore, the students who are remotely having elements similar to those who follow the class in person.

During class and outside it, the teacher proposes activities on the topics reviewed to complement their learning. These tasks as mentioned in architecture are based on active learning. Therefore, each of the activities evaluates an TLR to which the subject is governed. For example, if the TLR is “Implements data networks based on communication protocols and standards” the teacher raises an exercise where this development in student learning is evaluated. To do this, the exercise can be in a setting with communication teams, group work, concept maps, discussion forums, questionnaires, *etc*. The development of the proposed activities is evaluated in the LMS. The grades of the activities are stored in databases, as in all the systems that the teacher uses for the development of activities.

The data stored in the databases is processed by a Hadoop framework. The framework has been previously developed by the authors and is presented in the works (Iván Ortiz-Garcés & Facultad, 2019; Villegas-Ch, Palacios-Pacheco & Luján-Mora, 2019a), it allows a scalable data analysis that guarantees the results of structured and unstructured data. The processing framework looks for patterns in the data generated by the students. For example, if several students obtain grades less than 6/10, with six being the average grade with which a student passes the subject. The framework identifies these cases as anomalies and generates an analysis with greater granularity. This means that the system enters to review each grade obtained in each of the activities during a previously established period. The evaluation period is generally done in two partials during the semester. The system to identify the problem of the students reviews each activity developed by each student.

The processing stage also includes a recommendation system that uses AI to analyze the learning status of each student. The system identifies the factors that influence student learning. If you scored less than 6, present the results and provide feedback on the flow chart so that learners can adjust the activities or instructional method. On the contrary, if the student exceeds the grade point average, he presents the results to all those involved and stores the results that will be used in future effectiveness analyzes and exits the system.

In the first analysis, students are identified with problems in academic performance. In Table 1, the results of the analysis are presented, where the academic data, the data from the synchronous meeting system and the financial sources of 24 students of the networks I course were integrated. The relationship of the data is as follows; academic sources provide personal information of students; to present them an identifier has been placed for the proper management of the privacy of the information. The system contains the final grades of the students. The third column presents information that the framework crosses with the data from the synchronous meeting system. The system records the connection time the student has in minutes. In the audit, the system presents the effective time of each student during the session, this is done by detecting events that the student has with the peripherals of her computer. For example, the camera detects the presence of the student, the software detects the activities that the student has, and issues reports on this interaction. The keyboard and microphone are used to detect the student’s dedication while in the synchronous session. The academic period is made up of 16 weeks of which 14 are effective weeks of classes with three hours per week assigned to the subject evaluated. Two weeks used by the teacher to carry out comprehensive evaluations to students. Therefore, a student who effectively completes his synchronous classes will have 42 effective hours.

**Table 1:** Results of student grades by time of dedication and verification of financial problems.

Student	Qualifications	Dedication time	Income level
1	3	42	3
2	4	42	2
3	8	37	1
4	3	38	1
5	7	25	3
6	6	42	1
7	8	40	1
8	5	41	1
9	5	26	1
10	4	40	2
11	7	15	2
12	4	38	2
13	6	40	1
14	6	40	1
15	10	41	3
16	8	21	1
17	5	41	3
18	8	17	3
19	9	24	1
20	9	39	2
21	9	33	1
22	7	30	1
23	8	33	1
24	6	36	2

The fourth column presents the income level data; these data are obtained through surveys of students. The objective is to determine if there are financial problems for the students to generate the tuition payments. A weight was added to the answer to be able to calculate the correlation coefficient with the dependent variable:

Due to the pandemic, you have financial problems to cover tuition costs.

• High: 3
• Medium: 2
• Low: 1

From the results obtained, there is information that is easy to relate. For example, the majority of students who meet or exceed the average of 6 that allows them to pass the subject, exceed 33 h of effectiveness in classes. There is only one case that must be analyzed, since it exceeds the percentage of 6, but its effectiveness in classes reaches 17 h. There are eight students who do not meet the requirements to pass the course. These cases are analyzed in greater granularity to identify possible problems in learning. The first conclusion of the analysis determines that the grades have a direct relationship with the number of effective class hours that the student presents.

The next step is to determine the cause of the problem that is pushing students to perform poorly. To identify the problem, the LMS database is integrated. In this the information about the activities that the student carries out are recorded. Table 2 shows the details of the activities that the student developed in the first quarter. The activities are proposed by the teacher and respond to the needs and issues that the teacher decides to evaluate. In the Networks I course, students must fulfill two types of activities, the autonomous activities are exercises of network configuration on the topics that are developed during the synchronous meetings.

**Table 2:** Results of the activities proposed for the development of the first part of classes.

**Student**	Autonomous activities				Questionnaires				Exams			Total final
	**1**	**2**	**3**	**Total**	**1**	**2**	**3**	**Total**	**Prac**	**Teor**	**Total**	
1	1	0.5	0	0.5	2.5	2	1	1.8	0.2	0.5	0.7	3
2	1	1.25	0.5	0.9	1	1.5	2	1.5	0.6	1	1.6	4
4	1	0.8	0.3	0.7	0.3	1	0	0.4	0.5	1	1.5	3
8	1	1.25	0.5	0.9	2	1.5	2.5	2.0	0.6	1	1.6	5
9	2.5	2	1.7	2.1	1	1	2	1.3	1	0.1	1.1	5
10	0.75	2.25	1	1.3	2	1.5	1	1.5	0.4	1	1.4	4
12	2.5	1	1	1.5	0	1	0	0.3	0.5	2	2.5	4
17	2.5	1.5	2	2.0	0.1	0.6	0.5	0.4	2.3	0.5	2.8	5

Homework is graded out of 2.5 points and the first total takes the grade point average. Then there are the continuous assessment activities, identified as questionnaires. These activities evaluate the conceptual knowledge of the subject; they are quick questionnaires where students choose one or more options according to the question posed. The next column with the total label, takes the average of the questionnaires that have been raised during the academic period. The activities add up to five points and the remaining five points are evaluated in tests. Where the column Prac. It refers to the practical test and the Theor column. To the theoretical exam. The distribution of scores is three points for the practical evaluation and two points for the theoretical evaluation. The Final Total column corresponds to the sum of the activities and exams and is the one that determines whether a student passes the subject.

**Figure 8:**
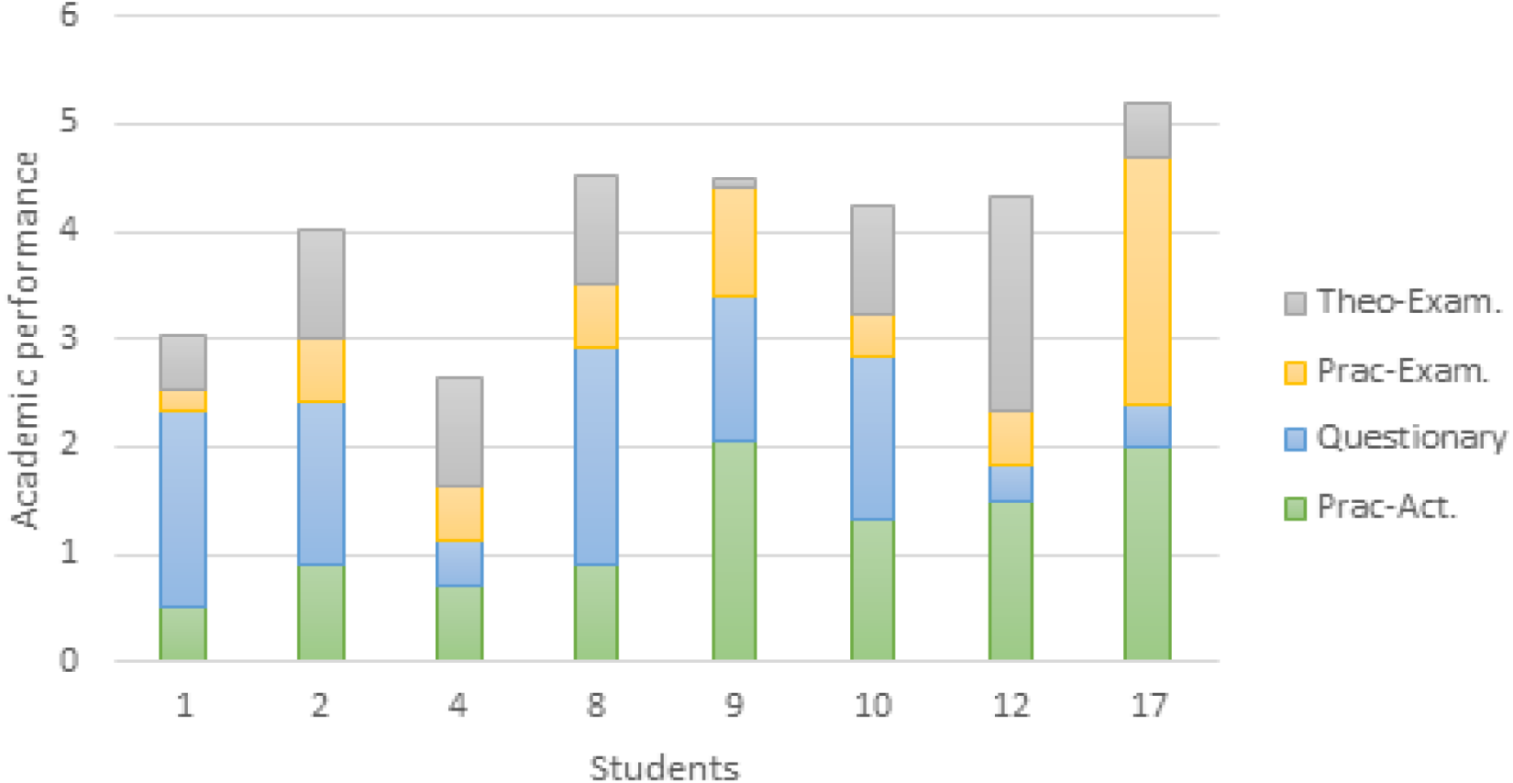
Graphical representation of the behavior of students’ grades.

In the results table the information is not clear, or it is difficult to determine what is happening in this analysis. Therefore, the Hadoop framework deploys several tools that allow to interpret this knowledge. In Fig. 8, the performance of the different activities per student is presented. In the student axis, the identifiers of the eight students being analyzed are identified. In this way, it can be determined that students 1, 2, and 8 have a better performance in the development of questionnaire-type activities. In cases like that of student 17, it can be observed that the grades obtained in the practical activities respond to what they obtained in the practical exam. The visualization allows knowing the performance of the students in relation to the activities they develop throughout an academic period and those in charge of educational quality can clearly identify the factors that affect learning.

In the data analysis, the patterns present in the academic data are identified. However, it is necessary to establish an AI model to determine the factors that have the greatest influence on student learning. In the preprocessing phase, the detailed structure is generated in Table 3, in this table the attributes and the data type of each of these are presented.

**Table 3:** Structure of attributes assigned to the generated analysis in the classifier.

Attributes	Type of data
Students (a)	Text
Income level (b)	Text
Frequency in the study (c)	Numeric
Environment (d)	Numeric
Educational resources (e)	Numeric
Interaction (f)	Numeric
Schedules (g)	Numeric
Padagogy (h)	Numeric
Academic average (i)	Numeric
Approves (j)	Text
Learning (k)	Numeric
Dependent variable (l)	Text

**Table 4:** Data table with the correlation between independent variables and the dependent variable.

**a**	**b**	**b−1**	**c**	**d**	**e**	**F**	**g**	**h**	**i**	**j**	**k**	**L**
Yes	Low	1	3	0	3	2	1	5	3	False	E	1
Yes	Medium	2	4	3	2	1	0	3	4	False	C	3
Yes	High	3	4	0	0	5	2	1	8	True	A	5
Yes	High	3	1	3	0	0	5	1	3	False	E	1
Yes	Medium	2	1	5	4	1	1	2	7	True	B	4
Yes	Medium	2	1	1	5	1	5	4	6	True	B	4
Yes	High	3	5	2	4	4	1	5	8	True	A	5
Yes	Medium	2	4	5	5	0	1	4	5	False	C	3
Yes	High	3	0	1	2	5	2	1	5	False	D	2
Yes	Low	1	4	2	1	4	3	0	4	False	D	2
Yes	Low	1	0	0	3	0	3	1	7	False	E	1
Yes	Low	1	4	3	1	3	5	2	4	False	E	1
Yes	Medium	2	0	1	5	1	1	1	6	False	E	1
Yes	High	3	3	5	0	1	5	5	6	True	B	4
Yes	High	3	3	2	3	5	2	4	10	True	A	5
Yes	Medium	2	3	1	2	0	4	0	8	False	E	1
Yes	Low	1	4	4	3	2	3	2	5	False	C	3
Yes	High	3	5	5	1	3	5	2	8	True	A	5
Yes	Medium	2	5	0	5	2	1	3	9	True	A	5
Yes	Low	1	3	5	1	2	3	4	9	True	A	5
Yes	Medium	2	0	5	5	1	1	3	9	False	C	3
Yes	High	3	2	4	5	1	2	2	7	False	C	3
Yes	Medium	2	2	5	2	3	3	4	8	True	A	5
Yes	Low	1	1	5	4	3	1	2	6	False	E	1
Corr_coef		0.43	0.44	0.24	−0.01	0.36	−0.04	0.50	0.65			

**Table 5:** Most significant attributes for learning state detection in a remote education model.

**a**	**b**	**C**	**i**	**k**	**l**	**n**
Yes	Low	3	2	5	3	E
Yes	Medium	4	1	3	4	C
Yes	High	4	5	1	8	A
Yes	High	1	0	1	3	E
Yes	Medium	1	1	2	7	B
Yes	Medium	1	1	4	6	B
Yes	High	5	4	5	8	A
Yes	Medium	4	0	4	5	C
Yes	High	0	5	1	5	D
Yes	Low	4	4	0	4	D
Yes	Low	0	0	1	7	E
Yes	Low	4	3	2	4	E
Yes	Medium	0	1	1	6	E
Yes	High	3	1	5	6	B
Yes	High	3	5	4	10	A
Yes	Medium	3	0	0	8	E
Yes	Low	4	2	2	5	C
Yes	High	5	3	2	8	A
Yes	Medium	5	2	3	9	A
Yes	Low	3	2	4	9	A
Yes	Medium	0	1	3	9	C
Yes	High	2	1	2	7	C
Yes	Medium	2	3	4	8	A
Yes	Low	1	3	2	6	E

Table 4 presents the academic data of the 24 students included in the analysis. To these data, he calculated the correlation in each of the 11 initial columns (independent variables) with respect to the last column called Dependent variable. In column “b−1” the values of the income level that refer to financial problems in the students are replaced, Low = 1, Medium = 2, High = 3; The change to a numerical data is carried out to determine the value of the correlation coefficient. For the calculation of the correlation, the values in the column “Dependent variable” are substituted as follows, *A* = 5, *B* = 4, *C* = 3, *D* = 2, *E* = 1.

In addition to the values of each attribute, the existing correlation coefficient with the dependent variable was added to the table. Values greater than 0.35 are the values closest to the trend line, therefore they are the most significant and decisive attributes in learning. These attributes are the income level, the frequency in the study, the interaction with the teacher, the pedagogy used in the remote classes and the academic average. Table 5 presents the five categories with the data used in the analysis. Where the column “a” indicates that they are students, and in this way their personal information is saved.

**Figure 9:**
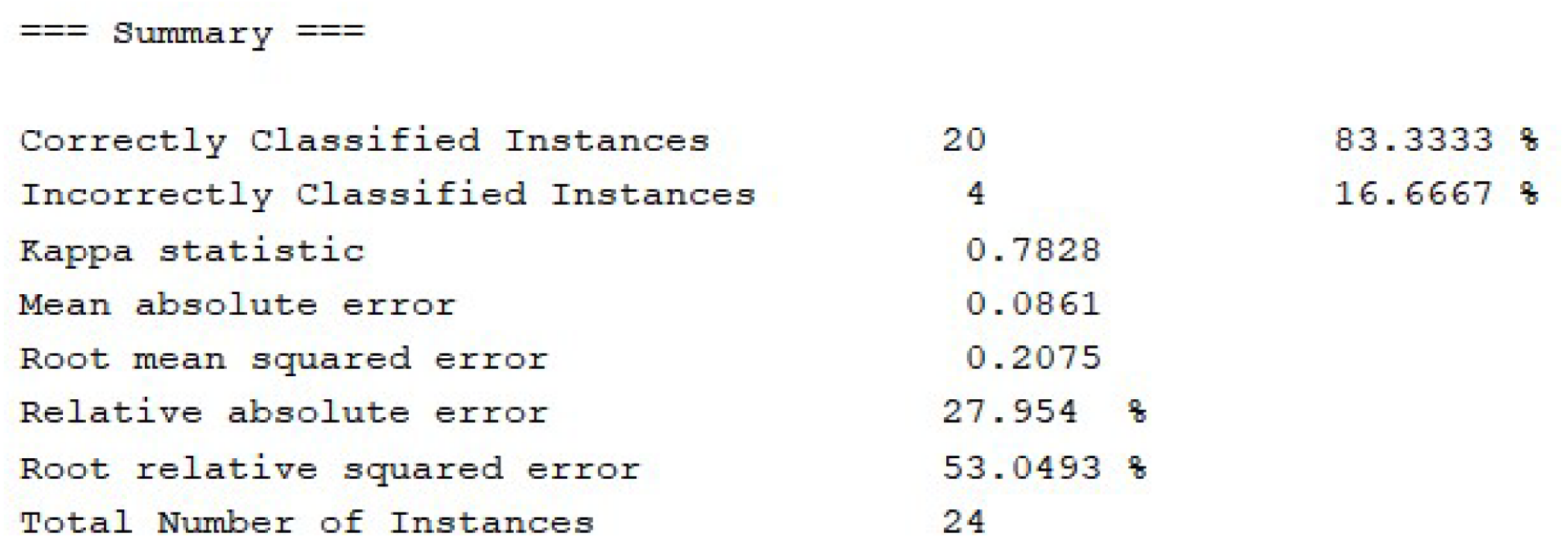
Summary of instance classification values.

The technique used for learning is through the J48 algorithm, in Fig. 9, the details of the analysis are presented. Of the 24 instances entered, 20 have been classified as correct with a percentage of 83.3333%, which is a high value. Adding a larger volume of data is expected to increase the efficiency of the algorithm.

**Figure 10:**
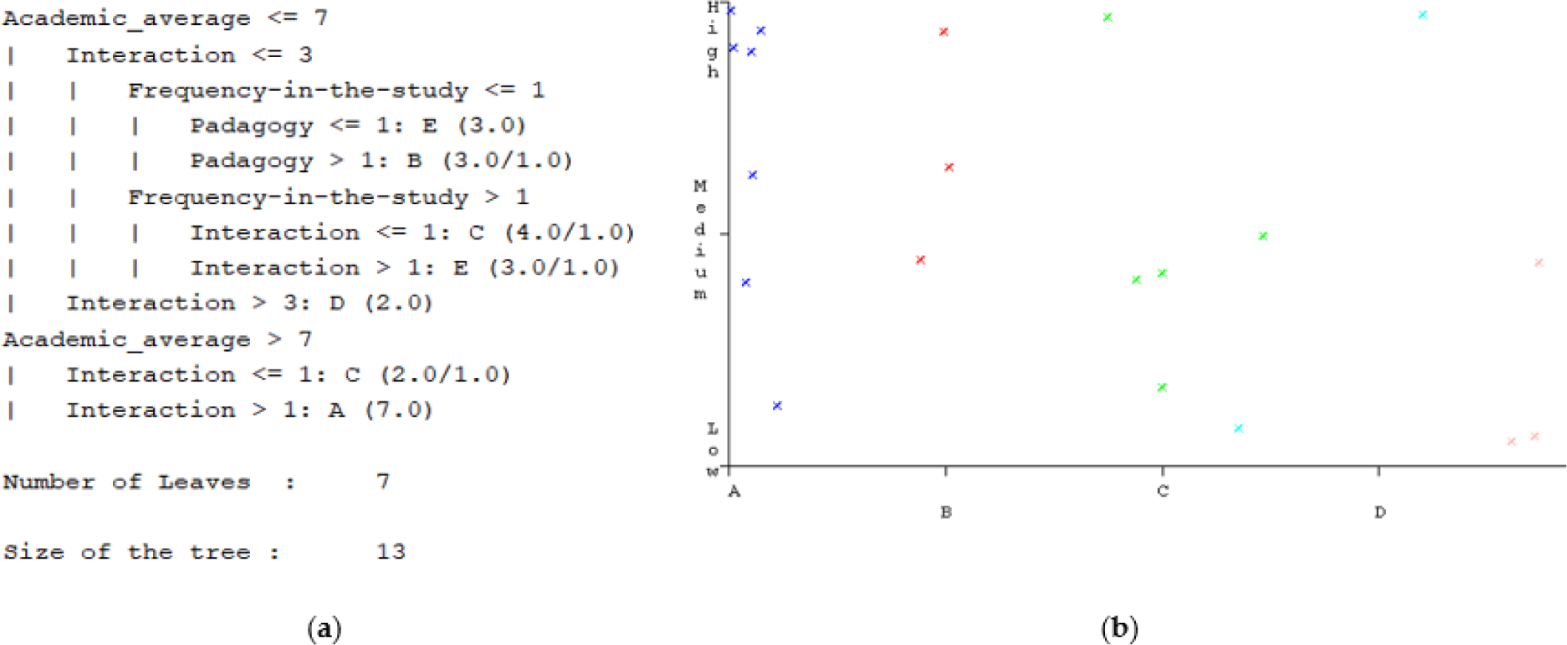
The figure presents decision tree information: (A) Describes the decision tree data generated by the J45 algorithm; (B) Present an example of the visualization of the factors that influence learning.

In Fig. 10A, the details of the tree generated by the algorithm are presented, it determines the levels and the size of the tree. It is necessary to consider that several of the factors that were considered in Table 2 were discriminated by the low values in their correlation coefficients with the student average. By this discrimination the values in the number of levels and the size of the tree have been obtained. At each level, it is observed how the different factors affect the performance of the students. For example, there are 3 students who are in group E, this being the students who have an average of 0–2; This means that they have lost the subject, but the factors that have the greatest incidence are pedagogy and interaction with the teacher. This problem has arisen and is very recurrent due to the change in educational modality. With these data, the academic quality area can make decisions to improve these factors.

In Fig. 10B, the visualization of another case is presented where the factor of financial problems is contrasted with the learning that is exposed as the averages obtained. In this, it is observed that seven students obtained grades between 8–10, the average with which they pass the subject. Of this group, 4 have high financial problems; this analysis has the greatest impact on groups that do not have a percentage higher than 6/10. These focus on group D and E, here it is important to establish that out of 9 students 4 have high financial problems. This being the factor in which the university must work with the highest priority to establish scholarships or financial aid that avoid an increase in dropout rates.

## Discussion

Learning goes through critical moments that have been exploited by the pandemic that affects society. Education has been forced to evolve and deliver projects that adjust to the reality of the students. Traditional educational models need to integrate ICT as a means to change their paradigm (Benshoff & Gibbons, 2011). Traditional models work in environments where all knowledge is based on the teacher. However, it has been shown that when the environment changes teachers are also affected and the line between learning and academic achievement is broken (Charles, 2006). This relationship is clearly evidenced in studies that have determined that the remote education model did not necessarily affect students’ grades, but it did directly affect learning. To solve these problems, it is necessary to integrate and create spaces where teachers and students coincide in a suitable environment for the generation of knowledge.

ICT have always been involved in education, specifically at the university level. But this is not always synonymous with effectiveness, for it to be an important change, it is necessary to modify the learning methodology and align it to the new needs of the students. Even considering that the remote education model does not include this change, since it simply changes the face-to-face classes for synchronous classes (Hill & Hannafin, 2001). This model has allowed the continuity of academic activities, but it has not done so efficiently. To do this, it is necessary to take the best of the face-to-face model and other educational models such as virtual or online education and apply them in remote education (Ali, 2020).

ICT generally accompany the student in the management of laboratories, LMS, educational projects. However, inclusion in academic and learning management has been very opaque. In this work, two fundamental pillars are taken at a technological level, such as ML with AI and Big data (Toquero, 2020). These tools create a robust assistant that works across students, teachers, and academic quality areas. Universities, having a large volume of data, can generate knowledge from the processing of this data. This is the point that this work addresses and exploits it to identify what the state of student learning is and then use the ML to validate this state and recommend activities that are centered on the student.

To reach this level, it is necessary to start with an architecture such as the proposal that starts from the traditional educational model and improves it in a scheme designed by capable. In this architecture, in addition, focusing on ICT is considered the necessary training for teachers to be successful in their activities. In training, the main point is to train the teacher for a progressive transition to an active learning model. By considering all the components exposed in our proposal, it is possible to improve learning and guarantee a decrease in devastating effects for education such as dropout.

This work is marked as a starting point for the inclusion of emerging technologies such as educational assistants. Where the needs of the students are identified through the analysis of the grades of the students in each partial or by activity. By clearly identifying problems, effective decisions can be made that quickly improve student learning. As has been done in the analysis and management of the recommendation system. According to the results obtained in Table 2, the application of this architecture is efficiently adapted to a remote model. As the architecture is scalable, the operation in the face-to-face education modality will improve considerably and reduce the burden on each teacher.

Other works analyze the causes and problems of remote education from a theoretical point of view, where the improvement of the educational model and learning depends strictly on the change of teaching methodology. Most of the work concludes that the academic load of the teacher becomes the main factor of failure in learning and the lack of methods such as active learning to do not allow the model to be optimal. With these conclusions, the recommendations they make are the migration to educational models that have been proven to be more effective, such as online education. However, our proposal welcomes the analysis carried out by these works and proposes a comprehensive solution that is based on the use of ICT (Lin et al., 2021; Stroeva et al., 2019). The basis of our ICT architecture is raised from a teacher support point of view, it does not load them with activities, it allows them to obtain clear information about the state of learning in each student. With this information, the AI can understand the weaknesses of the students and recommends a possible solution. Every result obtained from the analysis of educational data to the conclusions reached by architecture, it notifies both students and teachers. This helps stakeholders to take effective and efficient measures, thereby considerably reducing problems such as dropouts or repetition.

## Conclusions

ICT in the pandemic have demonstrated their functionality and potential so that organizations can give continuity to their businesses. Teleworking has prevented companies from staying in the market, and unfortunately, they have used them, they have disappeared or gone bankrupt. This panorama has been replicated in education and when considering that universities are organizations with greater complexity due to the number of users and the size of their structures, the problems multiply. To solve them, it has been necessary to create educational models that do not always depend on a physical place. Virtual and online education understood that distance, mobility and time are factors that can be overcome with the use of ICT. The opposite happens in face-to-face education, the need for a physical space and the dependence on teachers as the center of learning has compromised its effectiveness in the time that affects the COVID-19.

Universities that handle a face-to-face education model found it necessary to move to a remote model. These models are characterized by maintaining the entire concept and model of face-to-face education, but they execute it with synchronous classes. This way of executing it has not been the most appropriate and has generated many learning problems. What requires redefining the educational model, to do so requires the integration of ICT. But integration must become the architecture from which all educational components start. Emerging technologies such as AI, IoT and Big data become the ideal assistants to propose any student-centered architecture.

By having systems that oversee continuously monitoring students with the opportunity to create ideal environments for the generation of learning, an efficient educational model is created. To do so, an educational architecture based on ICT is proposed, which is based on data analysis. Identifying the state of learning in each student allows classifying it according to the patterns it presents. With this knowledge it is possible to generate activities focused on meeting the needs of each individual, it is even possible to reach a personalized education.

The integration of technologies such as Big data and artificial intelligence opens up a number of possibilities in the creation of digital environments that guarantee the resolution of problems. In this work, when implemented in an educational environment, they function as an ideal assistant who oversees student follow-up, reducing the teachers’ management time. Those who, having the information on the state of knowledge and the necessary time, can create activities and resources focused on the needs of the students. In addition, the time that is generally dedicated to identifying the cases where there are major deficiencies in learning, can be used to access training on the use of ICT and new virtual education methods. AI, every day has greater applications, in education its ability to generate knowledge from the analysis generated by Big data is useful to establish a guide that helps to improve the educational model. The ability of the system to understand what the learning situation of each student is, allows creating, even personalized education models. In addition, among the characteristics of our proposal, the availability to recommend a change in the activities or evaluation mechanism stands out, which leads to including in the architecture the management of laboratories and virtual environments where active learning can be applied.

The proposed architecture does not focus only on the student, it places him or her as the main actor, but proposes improvements that include the knowledge of the teachers. Any educational model that makes use of ICT requires the necessary knowledge of all those involved. If one of these actors fails, the component that is affected is learning. For this reason, it is necessary that each layer of the architecture be fully developed. In the results and as it is a proposal, the population that was considered has been very limited. However, it has been possible to evaluate the architecture in each of its stages. As future work, it is proposed to integrate a greater number of data sources and increase the volume of data managed by the Big data framework used.

Learning in a remote education model can be improved by integrating an ICT architecture like the one proposed. Even though this work considers a certain presence of teachers and students to generate suitable environments for learning, it is possible to adjust it to a totally face-to-face model or an online education model. To optimize it, the architecture must take greater emphasis on integration with active learning. In addition, it will be necessary to integrate new innovative technologies that contribute to the interest of students such as the IoT, Blockchain, Expert systems, *etc*. This approach is considered as future works where architecture is applied in an online education model in order to improve learning and its educational management.

## Supplemental Information

10.7717/peerj-cs.781/supp-1 Supplemental Information 1 Raw data

## Additional Information and Declarations

### Data Availability

The following information was supplied regarding data availability: The data downloaded from the LMS used by the university that participates in the study is available in the Supplemental File.

## References

[ref-1] Akopova E, Przhedetskaya N, Bogoviz AV, Ragulina J, Alekseev A (2019). Formation of remote education as a means of restoration of Russian recessive regions’ economy. International Journal of Educational Management.

[ref-2] Ali M, Hussein A, Al-Chalabi HKM (2020). Pedagogical agents in an adaptive E-learning system. SAR Journal - Science and Research.

[ref-3] Ali W (2020). Online and remote learning in higher education institutes: a necessity in light of COVID-19 pandemic. Higher Education Studies.

[ref-4] Almaiah MA, Al-Khasawneh A, Althunibat A (2020). Exploring the critical challenges and factors influencing the E-learning system usage during COVID-19 pandemic. Education and Information Technologies.

[ref-5] Alqurashi E (2016). Self-efficacy in online learning environments: a literature review. Contemporary Issues in Education Research (CIER).

[ref-6] Amory A, Seagram R (2004). Educational game models: conceptualization and evaluation. South African Journal of Higher Education.

[ref-7] Anbang X, Zhe L, Yufan G, Vibha S, Rama A (2017). A new chatbot for customer service on social media. Farmatsevtychnyi Zhurnal.

[ref-8] Bahasoan AN, Ayuandiani W, Mukhram M, Rahmat A (2020). Effectiveness of online learning in pandemic COVID-19. International Journal of Science, Technology & Management.

[ref-9] Beldarrain Y (2006). Distance education trends: integrating new technologies to foster student interaction and collaboration. Distance Education.

[ref-10] Benshoff JM, Gibbons MM (2011). Bringing life to e-Learning: incorporating a synchronous approach to online teaching in counselor education. The Professional Counselor.

[ref-11] Bestiantono DS, Agustina PZR, Cheng T-H (2020). How students’ perspectives about online learning amid the COVID-19 pandemic?. Studies in Learning and Teaching.

[ref-12] Boiy E, Moens MF (2009). A machine learning approach to sentiment analysis in multilingual web texts. Information Retrieval.

[ref-13] Brown SW, King FB (2000). Constructivist pedagogy and how we learn: educational psychology meets international studies. International Studies Perspectives.

[ref-14] Charles D (2006). Universities as key knowledge infrastructures in regional innovation systems. Innovation.

[ref-15] Daniel SJ (2020). Education and the COVID-19 pandemic. Prospects.

[ref-16] De Zubiría S, Ríos Angel M, Merani S (1999). Intervención pedagógica en proceso de pensamiento en alumnos con bajo desempeño académico. Fundación Alberto Merani. https://repositorio.idep.edu.co/handle/001/149.

[ref-17] Fedorenko EH, Velychko VY, Stopkin AV, Chorna AV (2019). Informatization of education as a pledge of the existence and development of a modern higher education.

[ref-18] Fernández-Gutiérrez M, Gimenez G, Calero J (2020). Is the use of ICT in education leading to higher student outcomes? Analysis from the Spanish autonomous communities. Computers & Education.

[ref-19] Gowensmith WN, Murrie DC, Boccaccini MT (2010). Face-to-face versus threaded discussions: the role of time and higher-order thinking. Law and Human Behavior.

[ref-20] Hill JR, Hannafin MJ (2001). Teaching and learning in digital environments: the resurgence of resource-based learning. Educational Technology Research and Development.

[ref-21] Hinostroza JE (2018). New challenges for ICT in education policies in developing countries: the need to account for the widespread use of ICT for teaching and learning outside the school. ICT-Supported Innovations in Small Countries and Developing Regions.

[ref-22] Hodges CB (2008). Self-efficacy in the context of online learning environments. Performance Improvement Quarterly.

[ref-23] Hodges CB (2013). Suggestions for the design of e-learning environments to enhance learner self-efficacy.

[ref-24] Hssina B, Bouikhalene B, Merbouha A (2017). Europe and MENA cooperation advances in information and communication technologies. Advances in intelligent systems and computing.

[ref-25] Huda M, Maseleno A, Shahrill M, Jasmi KA, Mustari I, Basiron B (2017). Exploring adaptive teaching competencies in big data era. International Journal of Emerging Technologies in Learning (iJET).

[ref-26] Ortiz-Garcés I, Nicolás Yánez N, Iván Ortiz-Garcés WV-C, Facultad (2019). Performance data analysis for parallel processing using bigdata distribution. RISTI - Revista Iberica De Sistemas E Tecnologias De Informacao.

[ref-27] Jahng N, Krug D, Zhang Z (2007). Student achievement in online distance education compared to face-to-face education. European Journal of Open, Distance and E-Learning.

[ref-28] Kerr P (2016). Adaptive learning. ELT Journal.

[ref-29] Li H, Liu SM, Yu XH, Tang SL, Tang CK (2020). Coronavirus disease 2019 (COVID-19): current status and future perspectives. International Journal of Antimicrobial Agents.

[ref-30] Lin P-Y, Chai C-S, Jong MS-Y, Dai Y, Guo Y, Qin J (2021). Modeling the structural relationship among primary students’ motivation to learn artificial intelligence. Computers and Education: Artificial Intelligence.

[ref-31] Literat I (2015). Implications of massive open online courses for higher education: mitigating or reifying educational inequities?. Higher Education Research and Development.

[ref-32] Madakam S, Ramaswamy R, Tripathi S (2015). Internet of Things (IoT): a literature review. Journal of Computer and Communications.

[ref-33] McHugh J, Cuddihy PE, Weisenberg Williams J, Aggour KSSKV, Mulwad V (2018). Integrated access to big data polystores through a knowledge-driven framework.

[ref-34] Medhat W, Hassan A, Korashy H (2014). Sentiment analysis algorithms and applications: a survey. Ain Shams Engineering Journal.

[ref-35] Palmer D, Fazzari S, Wartenberg S (2016). Defense systems and IoT: security issues in an era of distributed command and control.

[ref-36] Pattanayak S, Mohapatra S, Mohanty S, Choudhury T, Saini H, Sayal R, Govardhan A, Buyya R (2019). Empowering of ICT-based education system using cloud computing. Innovations in Computer Science and Engineering. Lecture Notes in Networks and Systems.

[ref-37] Pregowska A, Masztalerz K, Garlińska M, Osial M (2021). A worldwide journey through distance education—from the post office to virtual, augmented and mixed realities, and education during the COVID-19 pandemic. Education Sciences.

[ref-38] Salmerón-Manzano E, Manzano-Agugliaro F (2018). The higher education sustainability through virtual laboratories: the Spanish University as case of study. Sustainability.

[ref-39] Sidpra J, Gaier C, Reddy N, Kumar N, Mirsky D, Mankad K (2020). Sustaining education in the age of COVID-19: a survey of synchronous web-based platforms. Quantitative Imaging in Medicine and Surgery.

[ref-40] Stroeva OA, Zviagintceva Y, Tokmakova E, Petrukhina E, Polyakova O (2019). Application of remote technologies in education. International Journal of Educational Management.

[ref-41] Sun L, Fukuda T, Resch B (2014). A synchronous distributed cloud-based virtual reality meeting system for architectural and urban design. Frontiers of Architectural Research.

[ref-42] Sunasee R (2020). Challenges of teaching organic chemistry during COVID-19 pandemic at a primarily undergraduate institution. Journal of Chemical Education.

[ref-43] Tchoubar T, Sexton TR, Scarlatos LL (2019). Role of digital fluency and spatial ability in student experience of online learning environments. Intelligent Computing.

[ref-44] Toquero CM (2020). Emergency remote education experiment amid COVID-19 pandemic. IJERI: International Journal of Educational Research and Innovation.

[ref-45] Totkov G, Gaftandzhieva S, Pashev G, Atanasov S (2020). A system for modelling of processes for data accumulation and synthesis in higher education. TEM Journal.

[ref-46] Valtonen T, Kukkonen J, Kontkanen S, Mäkitalo-Siegl K, Sointu E (2018). Differences in pre-service teachers’ knowledge and readiness to use ICT in education. Journal of Computer Assisted Learning.

[ref-47] Verma C, Pandey R (2016). Big Data representation for grade analysis through Hadoop framework.

[ref-48] Villegas-Ch W, Palacios-Pacheco X, Luján-Mora S (2019a). Application of a smart city model to a traditional university campus with a big data architecture: a sustainable smart campus. Sustainability.

[ref-49] Villegas-Ch W, Palacios-Pacheco X, Luján-Mora S (2019b). Artificial intelligence as a support technique for university learning.

[ref-50] Villegas-Ch W, Palacios-Pacheco X, Román-Cañizares M (2020). An internet of things model for improving process management on university campus. Future Internet.

[ref-51] Villegas-ch W, Palacios-pacheco X, Roman-Cañizares M (2021). Applied sciences analysis of educational data in the current state of university learning for the transition to a hybrid education model. Applied Sciences.

[ref-52] Watson J (2006). Blending learning: the convergence of online and face-to-face education. Analytica Chimica Acta.

[ref-53] Zhou L, Wu S, Zhou M, Li F (2020). ¡¡School’s Out, But Class’ On¿¿, The largest online education in the world today: taking china’s practical exploration during The COVID-19 epidemic prevention and control as an example. SSRN Electronic Journal.

[ref-54] Хакимов H (2020). Reforming the education system in the conditions of a new stage of development. Архивнаучныхисследований.

